# Does inter-border conflict influence the views of task sharing among community health volunteers in Nigeria? A qualitative study

**DOI:** 10.1186/s13031-022-00472-y

**Published:** 2022-07-23

**Authors:** Luret Lar, Martyn Stewart, Sunday Isiyaku, Laura Dean, Kim Ozano, Caleb Mpyet, Sally Theobald

**Affiliations:** 1 Liverpool School of Tropical Medicine, Liverpool, UK; 2 Sightsavers, Nigeria Country Office, Kaduna, Nigeria; 3 Department of Community Medicine, University of Jos/Jos University Teaching Hospital, Zaria Road Post Office, P.O. Box 5388, Jos, Plateau State Nigeria

**Keywords:** Inter-border conflict, Task sharing, Community health volunteers, Nigeria

## Abstract

**Background:**

Volunteer community health workers are increasingly being engaged in Nigeria, through the World Health Organization’s task sharing strategy. This strategy aims to address gaps in human resources for health, including inequitable distribution of health workers. Recent conflicts in rural and fragile border communities in northcentral Nigeria create challenges for volunteer community health workers to meet their community's increasing health needs. This study aimed to explore the perception of volunteers involved in task sharing to understand factors affecting performance and delivery in such contexts.

**Methods:**

This was a qualitative study conducted in fragile border communities in north central Nigeria. Eighteen audio recorded, semi-structured interviews with volunteers and supervisors were performed. Their perceptions on how task sharing and allocation of tasks affect performance and delivery were elucidated. The transactional social framework was applied during the thematic analysis process to generate an explanatory account of the research data, which was analysed using NVivo software.

**Results:**

Promotive and preventive tasks were shared among the predominantly agrarian respondents. There was a structured task allocation process that linked the community with the health system and mainly cordial relationships were in place. However, there were barriers related to ethnoreligious crises and current conflict, timing of task allocations, gender inequities in volunteerism, shortage of commodities, inadequate incentives, dwindling community support and negative attitudes of some volunteers.

**Conclusion:**

The perception of task sharing was mainly positive, despite the challenges, especially the current conflict. In this fragile context, reconsideration of non-seasonal task allocations within improved community-driven selection and security systems should be encouraged. Supportive supervision and providing adequate and timely renumeration will also be beneficial in this fragile setting.

## Background

Africa has 25% of the global disease burden, but only 4% of the health workforce [1, 2]. In Nigeria, there is an average of 30 doctors per 100,000 population, and this varies within geopolitical zones (GPZ), with the lowest ratios in the northwest and northeast (4:100,000). The ratio is 9 doctors per 10,000 population in the northcentral geoplolitical zone [3]. The ratio for nurses and midwives is 100 and 68 per 100,000 population respectively, and again lowest in the northwest at 21 per 100,000 [4]. These human resources for health (HRH) are also inequitably distributed, with rural areas facing the largest shortages. Yet 50.48% of Nigeria’s population live in the rural areas [5], where the health needs are greater and the poverty index is worse [4, 6]. These geopolitical differences and health challenges are exacerbated by the ongoing conflicts in these areas.

In low and middle-income countries, task sharing is a solution to these HRH inadequacies; whereby less professionally qualified volunteer or paid community health workers (CHWs) are engaged to bridge the health system and poor rural communities [7, 8]. In Nigeria, the CHW programme was established and linked to the principles of primary healthcare following the Alma Ata Declaration, which is key to task shifting and community participation [9, 10]. According to the World Health Organization (WHO 2010), “task shifting is a process whereby specific tasks are appropriately moved from more professional health workers to those with shorter training and fewer qualifications” [11]. It is often used interchangeably with task sharing, which is structured around a team and needs-based approach to rationally distribute tasks among health workers [12]. Nigeria developed a National Task Sharing Policy that was implemented in 2014, to address the gaps in essential healthcare services [3]. The process involved consultations with individuals and organizations within and outside the health sector [12].

### Community healthcare worker performance frameworks

The vision for CHWs laid out in the Alma Alta Declaration has not been fully realised; yet CHWs are instrumental in the delivery of health services, particularly rural and remote regions of the country. There are several frameworks aimed at supporting and improving CHWs’ performance in relation to the communities and health systems in which they work. For example, the 5-spice model [13] developed by Partners in Health, a Boston-based non-government organization (NGO) consists of five components: supervision, partners, incentives, choice and education within the healthcare system and community [13]. Whilst the logic model analyses optimal CHW performance as a function of good quality programming, within robust health systems and communities, it also explores how these actors interplay between inputs, outputs and outcomes that promote good performance [14].

Another framework is the transactional process social framework developed by Kok and colleagues [15]. This framework analyses the holistic interaction of the context, community, and health system that CHWs and their supervisors interact within. Supervision is a key hardware component of the transactional framework. The software components include their interests, relationships and power dynamics that exist in the contexts and systems they work in [8, 15]. The interplay of this environment, the economy and health policies form the power relationships and dynamics that shape the motivation of CHWs to deliver their services. This broad, analytical transactional framework is based on existing models [13, 16, 17] and findings that have been used by others to validate the framework to support improvement of healthcare services through policy development [15, 16, 18]. These frameworks can also be employed in fragile and conflict affected contexts, although the evidence base related to their application is weak [19].

### Community health workers in conflict affected and fragile contexts

There is need to better understand the influence of conflict, crisis and insecurity on volunteer CHWs’ motivation, experiences, the roles they perform and the relevance of existing performance models within these contexts. Over the last decade (2001–2021), the political crisis related to ‘indigene’ rights and political representation in the Plateau State capital, Jos has developed into a protracted communal conflict affecting most parts of the State and neighbouring communities [20]. Bassa, in Plateau State (northecentral GPZ) shares boundaries with Bauchi (northeast GPZ) and Kaduna (northwest GPZ) States [21], which have experienced similar conflicts. Furthermore, the resurgence of conflicts with pastoral communities has affected the area, threatening the security of volunteer workers and activities [21]. In border and fragile settings, the process of sharing or allocation, as transactional activities, could be complicated by heightened tensions due to stronger ethic and religious differences, that in turn affect motivations or views about the role. Therefore, this study aimed to explore the perceptions, experience, and performance of CHWs in the context of task sharing within communities in Bassa, Plateau State in Northcentral Nigeria and potentially to illuminate workforce dynamics in similar situations where regions experience conflict.

## Methods

**Fig. 1:**
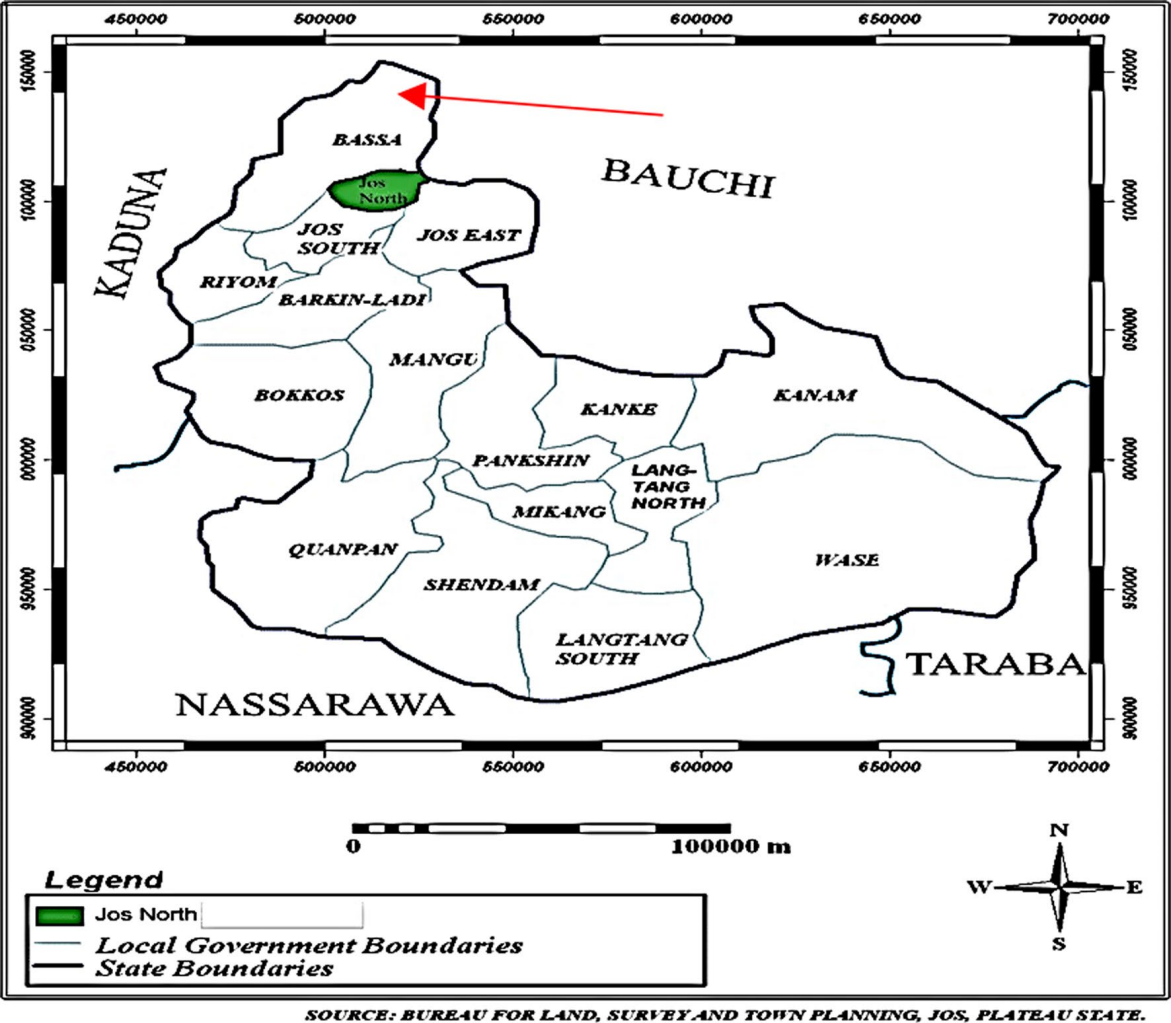
Map showing Plateau and surrounding states [25]. *Source*: Lekwot, Etal 2015

Volunteer CHWs and their supervisors’ experiences were best understood through the naturalistic lens of qualitative research [22], which is based on evidence grounded in the views of participants. We employed semi-structured interviews to understand CHW and facility heads’ views and experiences on performing their tasks and roles within a conflict affected area. Participants were drawn from six primary healthcare (PHC) centres across five rural communities of Bassa (Fig. 1). These communities have experienced recent ethnoreligious crises and share borders with two States that have also experienced such challenges. The study was conducted between April to September 2019.

### Participant characteristics

A pool of CHWs who reside in the community and provide volunteer services and the facility heads that allocate tasks to them was created [20] with the focal person at the health department of the local government area. Members of this pool were invited to participate in the study through phone calls. They were purposively and independently picked based on fulfilment of the study criteria of volunteering for at least a year and diversity in educational level and years of experience. Following informed consent processes, a total of 18 interviews including two facility heads, two assistant facility heads and 14 volunteer CHWs, were conducted by the lead researcher. After this point, there were recurring issues in the interviews, data saturation was likely to have been reached.

### Data collection

All participants agreed to come to the secretariat, which was a safe space where they said they felt comfortable commenting or criticising the system. The topic guides, which were translated into the local dialect and back translated into English had previously been piloted [23, 24]. The topic guides explored the following areas: tasks allocated, support, motivations, challenges faced, and current crises faced. Detailed explanation of keeping all information confidential, anonymising the information, and having separate interviews for the two groups (CHWs and their supervisors) helped to minimise any discomfort. It was explained to all participants that they were under no obligation to answer any questions which made them uncomfortable, or that they felt were too sensitive. A comprehensive security assessment was conducted with the Plateau State Ministry of Health, focal person and facility heads of the study area to ensure that the participants and researcher were safe. Thereafter, participants were contacted by phone and invited to be interviewed.

### Quality control/trustworthiness

The lead researcher was open about her positionality as a medical doctor, which helped to ensure that participants were aware of her background knowledge and the limitations of the health systems. The “bracketing off” method, which recognises the influence of positionality on the research process and outcomes [26] was deployed and use of a reflexive diary to document decisions taken, information around the iterative coding process, and emerging themes. At the end of each interview, the answers participants provided were summarised back to participants to check shared meaning through member checking [27]. A periodic audit trail, and discussion of the research process, was also conducted with co-authors at the Liverpool School of Tropical Medicine.

### Data analysis

The transcribed interviews were analysed inductively, using the framework approach and NVivo 12 software [22, 28, 29]. It provided a rigorous, but flexible basis for elucidating themes [29]. This allowed for situating findings within a larger discourse and making inferences of links between themes. The transactional framework was used to supoprt the production of an explanatory account of the research data based on emerging themes. It clearly shows the relationship between the health system, CHW performance and the factors that influence these within a much wider context.

## Results

To protect participant and location identity, pseudonyms were used (Table 1).

**Table 1:** Participant details

	Frequency	Percent
Sex		
Male	17	94
Female	1	6
Age category (years)		
0–27	2	11
28–55	16	89
Educational level		
Primary	**4**	22
Secondary	11	61
Tertiary	2	11
JCHEW	1	6
Marital status		
Married	17	94
Single	1	6
Occupation		
Facility head	4	22
Farmer	11	61
Student	1	6
Teacher	2	11
Years of experience as a CHW/supervisor		
0 < 5	1	6
5– < 10	5	28
10 < 15	5	28
15+	7	38
Health centres		
Public PHCs	18	100
Private PHCs	0	0

Three key themes emerged from our analysis given the inducive approach taken. (1) narratives on how the health system works (including sub themes on the selection and role of CHWs and the role of supervisors and role allocation) (2) barriers to task sharing (including barriers to performancebarriers associated with community and household conflict, the farming season and system hindrances); and (3) enablers including intrinsic motivation and community development.

### Theme 1: The community health system in context: how the health system works

#### Selection and the role of CHWs

Study participants (CHWs and facility heads) described how CHWs are selected by communities, with the involvement of relevant community stakeholders such as men, women, youth, and religious leaders. This selection is normally completed in collaboration with the existing health system and specifically the community head. However, a participant mentioned how selection processes, especially within border states, have been influenced by ethnoreligious crises.” *You know we will have to put our own in these locations…*” ***(Male, CHW Supervisor, 15*+ *years of experience)****.* The CHWs selected are generally dependent on the type of community and nature of the tasks.This is because communities have ther various health needs and sociocultural norms and recently the ethnoreligious crises have further narrowed the scope of CHWs that are selected. However, those of a certain ethnic and religious group are selected, whilst certain groups are left out of the selection process, due to fragile circumstances associated with the inclusion of non-indigenes who do not belong to the predominant religious group in that community.

The range of tasks performed by all CHWs were mainly promotive and preventive and none had purely curative or rehabilitative roles. Health promotion tasks included awareness creation including school-based deworming, mosquito net distributions, immunizations (especially polio). “*I tell the district head about the distributions and he calls the community members to inform them…*” (***Male, CHW, 15*+ *years of experience).*** Some also focused on personal and environmental sanitation. Preventive tasks included distributing medicines, nets distributions, immunizations and very rarely (in one participant), school eye screening. There was more priority placed on communicable diseases control.

Most participants described that gender intersects with other characteristics to shape selection processes or perceptions of the role of CHWs. However, one participant stated that lower literacy levels among females and their domestic demands limit their selection as volunteers.

“Yes, sometimes they pick equally, but sometimes we the males are more compared to the females. Sometimes you get to see that the woman is pregnant or has a newborn and sometimes lacking a cook for her children, so that alone leaves her restricted.” **(Male CHW, 5- < 10 years of experience).**

Another participant gave reasons for more male selections “*…because when we were distributing Mectizan…it is the males who read in the past and most women are not literate…*”* (****Male, CHW, 15*+ *years of experience).***

#### The role of supervisors and task allocation

Supervisors are trained on tasks which they cascade to CHWs so they can do their roles which as outlined above are mainly focused on prevention and promotion of communicable diseases such as malaria, schistosomiasis, soil transmitted helminths and polio. Supportive supervision strctures were limited, and attitudes were mixed with some seeing supervision as either absent or punitive, for example “*When we commence the work, we are with our supervisors…. If we make a mistake, they point it out, …*” ***(Male, CHW, 15*+ *years of experience).***

“I have never had any supervisor come to see my work since I started volunteering.” **(Female, CHW, 15+ years of experience).**

The task allocation process begins with training of the supervisors by the local government staff, and the supervisors then train the CHWs. The state implementers are responsible for this above the LGA level. Tasks are then collectively allocated to the CHWs by the supervisors, who rarely provide supportive supervision. Reports are then initially written by the CHWs and sometimes reviewed by supervisors and submitted, which is a rare, shared task amongst CHWs.

“I also encourage the teachers to send timely reports because if they do so, their work is complete. I also inquire that the report has been sent.” **(Male CHW, 10 < 15 years of experience).**

The task allocation process is clearly structured in terms of selection of volunteers, preparing them and their supervisors for the tasks, which further links the community with the health system. The views on its effectiveness were mixed**.**

### Theme 2: Barriers and enablers to task sharing

#### Barriers toperformance

This theme looked at circumstances, physical and intangible barriers that limit effective task performance and allocations. Sub themes emerged around community and household conflict, the farming season and system hindrances.

#### Community and household conflict

Facility heads and CHWs were both faced with barriers in allocating and undertaking tasks in areas affected by conflict. A participant described the decline in community participation in health activities due to conflict stating that “*but when we started this thing in 2007 to 2010 the community have been supporting but now the community are not supporting at all from the crises we experience, especially due to ethnic and religious differences.*” (***Female, CHW Supervisor, 15*+ *years of experience).***

Barriers to CHWs participation in conflicted affected areas included personal choices and attitudes, ethnoreligious background and trust. The quote below summarizes the common perceptions regarding trust that may be exacerbated by ethno- religious viewpoint and linked to national legislation and the right to voluntary decisions.“*Sometimes during polio, if we get to … communities and say *“*Asalam alakum*” *(the Muslim greeting), …, they tell us to stay outside and sometimes they say they will not give their children…*” ***(Male, CHW, 5–* < *10 years of experience****).*

All participants proferred solutions to overcoming challenges without being prompted. For example, when medicines are administered in communities by the CHWs and refusals from the community occur, the CHWs discuss the reasons why these medicines are necessary to promote healthy individuals and communities. If communities still refuse to take the medicines, despite explanations of benefits of doing so by the CHWs (which appeared to be a common experience), the focal person and the district head are informed.

At the household level, a participant linked barriers in his participation to his gendered role within his family. He highlighted their dissatisfaction with him abandoning family needs for the community, which was seen as not providing much gain. Another participant also shared similar views regarding this. “*…Challenges may come from your wife or children saying you have refused to do what will help the home, but something that is not beneficial.*” ***(Male, CHW, 10* < *15 years of experience).*** This was a recurring challenge from family members, and amplified in contexts of poverty.

#### Timing of task allocation

Some participants mentioned the rainy season as being a hindrance to them and coinciding with peak periods of medicines and net distributions, leading to community inaccessibiliy, increased attrition rates and negatively affecting farmwork. This was linked to the broader economic challenges they face.

“… Sometimes I don’t go to the farm… and when distributing drugs I can’t go to the farm except I give money to people that will go to farm for me,…” (**Male CHW Supervisor, 10 < 15 years of experience).**

This contributed to CHW attrition as emphasised by a participant***;*** “*Some have stopped because of this.*” ***(Male, CHW, 5–* < *10 years of experience).*** The majority of CHWs suggested the need for a change in timing of campaign based activities to support them to fulfil their role in this task shifting activity.

#### Financial and logistical limitations

The financial remunerations increased over the years and CHWs were paid in cash ($6.30 naira equivalent; 3500 per task allocation round) in 2018. However, this has currently changed to bank payments which has resulted in delayed or no payments to the CHWs, as most of them do not have bank accounts. Those who have them, travel to urban areas where the banks are, which can bring additional security challenges. They spend transportation costs and are left with little, as bank charges are deducted. A facility head mentioned this and how the current dwindling support led to attrition.

“…,CDDs what they are giving them the community is not supporting them thinking that the NGOs are supporting them, but the NGOs is only the transport allowance that they are giving them…” (**Female, CHW Supervisor, 15+ years of experience).**

A participant mentioned scarcity of commodities as a major system challenge. This also affected his own household as he forfeited his net for a community member, showing some of the sacrifices CHWs make. …“*I was given 100 nets… I initially kept one for myself, but an elderly man said he did not get one, so I gave him my own…*”* (****Male, CHW, 15*+ *years of experience).***

Logistic challenges related to transportation and financing were also mentioned by most participants. Some of them incurred out of pocket spending, as a result. Additionally, a robust system for logistics does not exist. “*When we distributed nets, we used my brother’s motorcycle and went house to house using our own money. We did not receive any money for that…*” ***(Male, CHW, 10–* < *15 years of experience).***

The need for more structured and stable incentivisation was a clear theme and a common view amongst both supervisors and CHWs.

### Enablers

#### Intrinsic motivation

Clear strong themes here included the intrinsic motivation among CHWs to volunteer and attributing most of their rewards to blessings from God, despite the often unsafe contexts that they work in. A participant expressed this as, “*…I am happy to do this because of the recognition I get in the community. Some think I am a health worker, and this makes me happy…, I am well respected.*” ***(Male,CHW, 15*+ *years of experience).*** Support from supervisors motivated them the most, despite the challenges they faced by living and working in conflict contexts.

A participant emphatically mentioned that **“***I am happy that you came because if we are doing this work and are being called and asked questions like this, it is good… This has not been done here since I started.*” (***Female, CHW Supervisor, 0* < *5 *years of experience).** This emphasises how CHWs (unlike in many contexts) have not participated in research, and have limited opportunities to share their expereinces and the challenges they face in these fragile contexts.

#### Community development

Partner support through financial support and the provision of medicines, bed nets among other commodities that CHWs distribute to their communities, was a powerful sentiment enabling most volunteers. A CHW had this to say…” *the medicines are donated to us from the foreign nations. They told us the value in foreign currency and if we are to convert it to our own, it will be a lot of money…*”* (****Male, CHW, 5–* < *10 years of experience).***

## Discussion

**Fig. 2:**
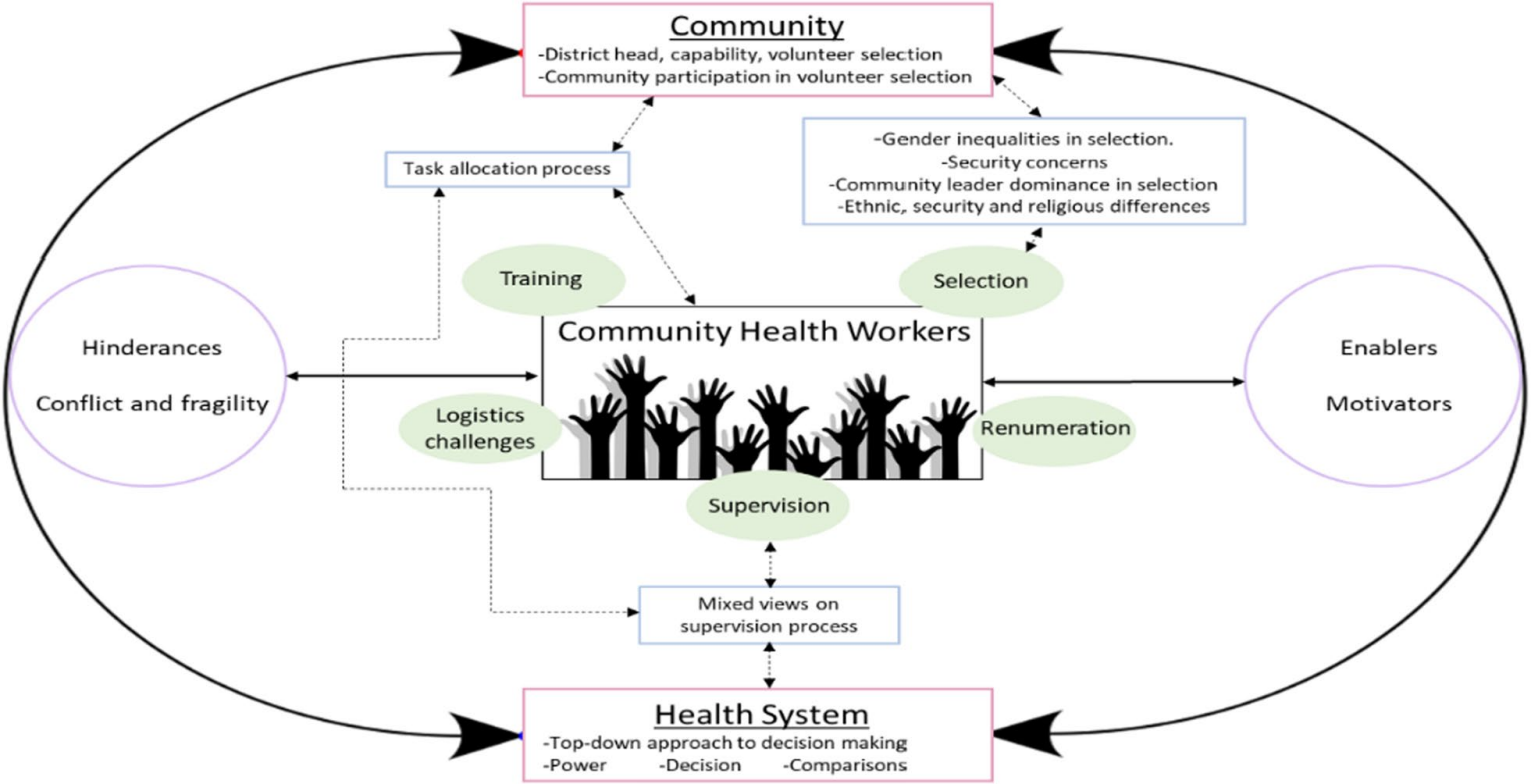
Illustration of key discussion points in relation to Kok et al.’s (2017) transactional framework process (adapted by authors)

Our study illuminates how CHW performance is a transactional social process shaped by context, system and intervention hard and software [8, 15]. We adapt Kok et al.’s model (2017) in Fig. 2 below to illustrate how community health systems in the the context of fragile border regions, (where there are ethnic and religious differences) in Nigeria are in turn shaped by interests, relationships and power dynamics that exist in the contexts and systems. These shape selection of CHWs, their role allocation, the supervision process and the barriers and enablers they face in their day to day work.

### System software and hardware: power, values, norms, trust and selection of volunteers; task allcoation and supervision

#### Volunteer selection

All participants agreed on the need for a transparent selection process that promotes gender equity, ability and that community participation supports CHWs. However, some role and literacy related gender and ethinic/relgious inequalities in selection emerged confirming studies in other contexts. In Nigeria, there were “silent” concerns about a truly transparent selection process due to security concerns, [30]. Furthermore, as reflected in studies elsewhere (Afghanistan) community leader “dominance” in the selection process could introduce bias in selection [31].

#### Task allocation

According to the WHO, the starting point for an effective design of CHW programmes is a sound situation analysis of population needs, health system necessities and resource allocation [32]. This may explain the focus of supporting partners and the mainly promotive and preventive tasks that CHWs are trained on and expected to undertake; and is also in accordance with national guidelines [3, 4]. The focus on tasks which are promotive and preventive, including community sensitization, mobilization and medicines distributions is in line with Perry et al.*’s*, [33] analysis of CHWs core tasks. In all 36 states in Nigeria and the Federal Capital Territory, one of the priority areas is combatting malaria [3], hence most CHWs performed these tasks, and others linked to national priorities.

#### Supervision, power and decision making: linking volunteer CHWs and the health system

The presence of some supportive supervision and cordial working relationship among participants emerged from the analysis. Community support was invaluable, as they were familiar with each other and the trust, values and community norms that they share. However, there were mixed views, on supervision and the gaps in the supervision process was mainly around attitudes of the CHWs to the allocated tasks.CHWs perceived that they are not fully involved in decision making, which they felt was made for them by their supervisors.

These findings differ from other studies [2, 24]. In a Mozambiquan study, CHWs felt that supervision was more fault finding than supportive. The supervisors also had no training and felt the workload of providing clinical facility-based services and community supervision was challenging [8, 34]. Again in a sysematic review, the need for a balance between the community and health facility was emphasised [35]. In a study conducted in Mkuranga District of Tanzania, the community were unaware of the CHWs [36], showing a gap in the link between and within all components of the transactional framework [37]. In our study, supervision as a key hardware component of the transactional framework [38], shaped CHWs on allocated tasks and enabled their performance in this study.

#### Enablers, motivators, barriers and their interlinkages

The community, economy and health system comprised the environment that enabled, motivated or created barriers for effective performance in this study. The interplay of themes and subthemes in these crucial determinants of performance among participants showed the interrelated, yet contradictory relationships between them. An enabler may simultaneously be a motivator and demotivator. For instance, financial partner and community support enabled and motivated as well as demotivated participants, when it was adjudged as inadequate, untimely, or as paid via inconvenient means. This links to debates in the literature around incentivising volunteer CHWs and the importance of the provision of stable financial remuneration [8, 39–41]. Power in decision making regarding mode of payment of CHWs by the system was a major concern for the supervisors.

In several studies, including one conducted among supervisors in Durna, South Africa, and with CHWs in Bangladesh, financial incentives did not demotivate CHWs to continue providing services, whilst reocognising the importance of collaboration between the community and health sector [34, 42–45]. Task allocations in the rainy season hindered most participants, who are farmers from farmwork. This is a recurring system challenge that could exacerbate the attrition rate.

Although majority of the participants willingly volunteered, they collectively attributed it to divine and national patriotic reasons, which is a common finding among the religious Nigerian population and has been identified in other contexts too, e.g. Bangladesh, Ethiopia, Indonesia, Kenya, Malawi, Mozambique Nepal, and Tanzania  [46–48].

#### Implications for the context, conflict, fragility and the rest of Nigeria and beyond

The National Task Sharing Policy should be reviewed in collaboration with the volunteer CHWs and their supervisors with more emphasis on broader task allocations in line with current priorities, remuneration, integration and clear task allocation process. This will ensure that their voices are heard. Furthermore, the varying contexts, ethnic, religious and security situations CHWs work in should be acknowledged within the policy with clearly stated strategies on how to tackle such challenges. Challenges of CHWs are similar to existing underlying challenges, and dwindling community support (found in many contexts) but are also shaped by fragility and security challenges. Priority areas to support this criticial cadre should include seasonal considerations, accessibility, and a clearly defined incentive package, which was the main concern of all participants in this study, following the current security challenges.They will need additional resources for better planning, in case of unforeseen challenges that these conflict contexts may create for them, their families and the communities that they live and work in. Dialogue with community leaders and training of staff on health delivery in interborder conflict will be beneficial.

#### Study strengths and weaknesses

The main strength of this study was the inclusion of participants from a variety of communities sharing borders with two states to explain their perceptions and compare them with their supervisors’. Hence, the study contributes to developing a community and health systems-based approach to task sharing studies within fragile contexts. The main weakness was that data collection did not include beneficiaries of these services provided by the implementers. This was because of the existing security challenges in the study sites, which limited travel to physically collect this data. Including community perspectives would have been illuminating and could be the focus of future research. Relatively few female CHWs were recruited into the study.

## Conclusion

This study showed that promotive and preventive tasks are performed by CHWs, through an established task allocation process, linking the communities with the health system. CHWs work in these fragile and rural communities is challenging, with recurring crsies, ethno religious unrests and medicine and bed net distributions were undertaken through risky terrains. Understanding CHWs perspectives is critical: they were motivated by a complex interplay of extrinsic and intrinsic motivation and demonstrated personal commitement and zeal to provide services in challenging circumstances. More collaborative, multidisciplinary relationships and research with implementers at community levels and the health system could facilitate co-creation of interventions such as collective microplanning, development of more robust security cover for implementers and a gender sensitive implementation process that can serve as advocacy toolkits for similar fragile and conflict contexts to learn from.

## Data Availability

All data and materials used for his study can be accessed upon request from the corresponding author.

## Abbreviations

CHW: Community health worker
HRH: Human resources for health
NGO: Non-governmental organization
WHO: World Health Organization
